# Enhanced Ghrelin Levels and Hypothalamic Orexigenic AgRP and NPY Neuropeptide Expression in Models of Jejuno-Colonic Short Bowel Syndrome

**DOI:** 10.1038/srep28345

**Published:** 2016-06-21

**Authors:** Laura Gillard, Lore Billiauws, Bogdan Stan-Iuga, Lara Ribeiro-Parenti, Anne-Charlotte Jarry, Jean-Baptiste Cavin, Françoise Cluzeaud, Camille Mayeur, Muriel Thomas, Jean-Noël Freund, Jean-Marc Lacorte, Maude Le Gall, André Bado, Francisca Joly, Johanne Le Beyec

**Affiliations:** 1 Inserm UMR1149, UFR de Médecine Paris Diderot, Université Paris Diderot, Sorbonne Paris Cité, DHU Unity, AP-HP, F-75890 Paris, France; 2 AP-HP, Hôpital Beaujon, Service de Gastroentérologie et d’Assistance nutritive, Clichy, France; 3 AP-HP, Hôpital Bichat - Claude Bernard, Service de Chirurgie Générale et Digestive, F-75018 Paris, France; 4 Micalis Institute, INRA, AgroParisTech, Université Paris-Saclay, 78350 Jouy-en-Josas, France; 5 INSERM UMR_S1113, Université de Strasbourg, Faculté de Médecine, FMTS, 67081 Strasbourg, France; 6 INSERM, UMR_S 1166, Research Institute of Cardiovascular Disease, Metabolism and Nutrition, ICAN, Université Pierre et Marie Curie, Sorbonne Université, F-75013, Paris, France; 7 AP-HP, Hôpital Pitié-Salpêtrière-Charles Foix, Biochimie Endocrinienne et Oncologique, F-75651, Paris, Cedex; 8 Université Pierre et Marie Curie, Sorbonne Université, F-75005, Paris, France

## Abstract

Short bowel syndrome (SBS) patients developing hyperphagia have a better outcome. Gastrointestinal endocrine adaptations help to improve intestinal functions and food behaviour. We investigated neuroendocrine adaptations in SBS patients and rat models with jejuno-ileal (IR-JI) or jejuno-colonic (IR-JC) anastomosis with and without parenteral nutrition. Circulating levels of ghrelin, PYY, GLP-1, and GLP-2 were determined in SBS rat models and patients. Levels of mRNA for proglucagon, PYY and for hypothalamic neuropeptides were quantified by qRT-PCR in SBS rat models. Histology and immunostaining for Ki67, GLP-1 and PYY were performed in SBS rats. IR-JC rats, but not IR-JI, exhibited significantly higher crypt depths and number of Ki67-positive cells than sham. Fasting and/or postprandial plasma ghrelin and PYY concentrations were higher, or tend to be higher, in IR-JC rats and SBS-JC patients than in controls. *Proglucagon* and *Pyy* mRNA levels were significantly enhanced in IR-JC rats. Levels of mRNA coding hypothalamic orexigenic NPY and AgRP peptides were significantly higher in IR-JC than in sham rats. We demonstrate an increase of plasma ghrelin concentrations, major changes in hypothalamic neuropeptides levels and greater induction of PYY in SBS-JC rats and patients suggesting that jejuno-colonic continuity creates a peculiar environment promoting further gut-brain adaptations.

Short bowel syndrome (SBS) results from extensive resection of the small intestine. It is the leading cause of chronic intestinal failure and is treated principally by parenteral nutrition (PN). The degree of malabsorption depends on both the extent of the resection and the anatomy of the remnant bowel. Adaptive phenomena occur during the two to three years following the surgery and are characterized by improvements in intestinal absorption^1^,^2^, increase of hormonal secretion^3^,^4^, development of an hyperphagia^2^,^5^ and dysbiosis of the gut microbiota^6^,^7^,^8^. These changes, which are highly variable and unique to each patient, may facilitate weaning off of PN. Oral intake plays a major role in these adaptations^9^ as does the presence of the terminal ileum and/or colon.

Gut hormones are key factors in this spontaneous intestinal adaptation^10^. Increased secretions of glucagon-like peptide-1 (GLP-1) and glucagon-like peptide-2 (GLP-2) have been reported in preclinical models^11^,^12^ and in SBS patients with the colon in continuity^3^, but not in SBS patients with jejunostomy (i.e. with no colon and no ileum)^4^,^13^ These hormones are secreted, together with Peptide YY (PYY), by the enteroendocrine L cells of the ileum and colon, in response to nutrient stimulation^14^. They play key roles in orchestrating gastrointestinal functions, such as intestinal trophicity^12^, expression of intestinal nutrient transporters^10^,^15^,^16^ and gastro-intestinal motility^4^,^17^. The role of GLP-2 in these intestinal functions has been largely documented^18^ in SBS patients and its therapeutic use (in the recombinant form of Teduglutide) has now been approved in adults with SBS dependent on PN. At last, PYY and GLP-1 are considered to be molecular mediators of the ileal brake^19^. They inhibit gastric emptying and intestinal transit, thereby increasing the time for the nutrients to be absorbed by the intestinal mucosa.

The development of hyperphagia, which increases over time in SBS patients after reestablishment of colonic continuity^2^, makes it possible to decrease dependence on PN by increasing net nutrient absorption^2^. It is associated with a good prognosis for the weaning off of PN^2^,^20^. The molecular and physiological mechanisms underlying the development of this compensatory overeating in SBS patients remain unknown. In addition to their gastro-intestinal effects, gut hormones (such as CCK, PYY, GLP-1 and ghrelin) participate in the regulation of appetite and food intake^14^. Ghrelin, is the unique orexigenic gut hormone^21^,^22^. Plasma ghrelin levels increase before meal, stimulating hunger and meal initiation, and then decrease after the meal^21^. These peripheral gastrointestinal signals are integrated into the hypothalamus, which contains two populations of neurons: orexigenic neurons co-expressing neuropeptide Y (NPY) and agouti-related peptide (AgRP) and anorexigenic neurons co-expressing pro-opiomelanocortin (POMC) and cocaine- and amphetamine-regulated transcript (CART)^14^,^23^ Ghrelin increases food intake through the activation of hypothalamic orexigenic neurons^24^. Little, if anything, is known about the expression of these neuropeptides in preclinical models of intestinal resection. Only one study has reported data for the fasting concentrations of ghrelin and PYY in SBS patients with adaptive hyperphagia but their postprandial levels were not evaluated^25^. Finally, the measure of circulating gut hormones is recognized of great interest for the follow-up of patients with gastrointestinal pathology and/or surgery. However their baseline and postprandial concentrations are rarely evaluated in combined panels in SBS patients with or without ileum nor compared with control subjects and little is known about what the colon or the ileum specifically brings to intestinal endocrine adaptation in SBS.

To address the colonic cellular and molecular mechanisms of adaptive hormonal secretions, we set up two rat models of extensive intestinal resection with colon in continuity and with or without a resected ileum. We use these models to analyze food intake, hypothalamic neuropeptides expression and to evaluate gastro-intestinal production and secretion of gut hormones (ghrelin, PYY, GLP-1 and GLP-2) in comparison with sham-operated animals. We further analyzed the clinical relevance of hormonal adaptation in relation to food intake (fasting and post-prandial gut hormone secretion) in jejuno-colonic and jejuno-ileal SBS patients.

## Results

### Preclinical models

#### Characteristics of rat models

**Figure 1:**
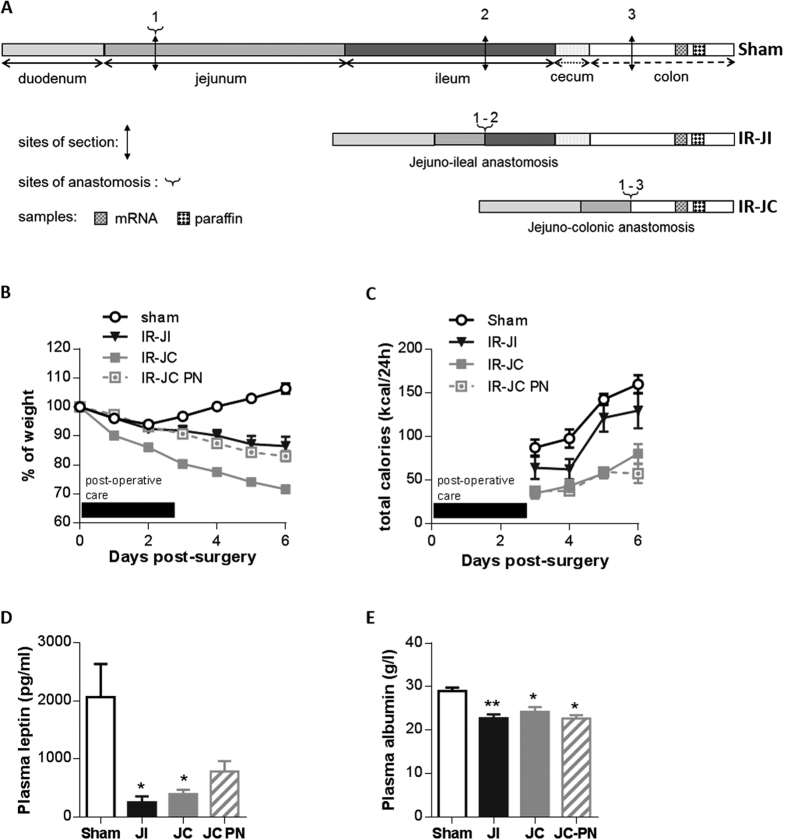
Body weight loss, plasma leptin levels and histological adaptation of the remaining colon mucosa after intestinal resection in rats. (**A**) Schematic representation of the surgeries and colonic tissue collections at day 7 post-surgery. Length of resected or remnant part of intestinal segments are not proportional. (**B**) Comparative time-dependent body weight loss represented as percent of the preoperative weight and (**C**) total calorie intake (oral intake and parenteral nutrition) in IR jejuno-ileal (IR-JI), IR jejuno-colonic (IR-JC), IR jejuno-colonic PN (IR-JC PN) and sham-operated (Sham) rats during 7 days after surgery. Black box corresponds to the period of the postoperative care. (**D,E**) Plasma levels of leptin (**D**) and albumin (**E**) in the same groups. Data are represented as mean ± SEM of n = 6 for sham, n = 4 to 6 for IR jejuno-ileal, n = 10 for IR jejuno-colonic, n = 5 to 6 for IR jejuno-colonic with PN. *P < 0.05, **P < 0.01, vs sham-operated rats based on non-parametric Kruskal-Wallis test followed by Dunn’s adjusted multiple comparisons.

All rats were on oral nutrition with free access to food, but a group of IR-JC rats received also PN to partially compensate for their malabsorption. Seven days after surgery, sham rats had gained 6% of their pre-operative weight, whereas IR jejuno-ileal (IR-JI) and jejuno-colonic (IR-JC) rats had lost 14% and 29% of their pre-operative weight, respectively (Fig. 1B). IR jejuno-colonic rats under enteral and parenteral nutrition (IR-JC PN) had lost 17% of their pre-operative weight without increasing their food intake suggesting that PN (12 kcal/day) was beneficial for the nutritional status of the IR-JC rats. Total calorie intake (oral intake and parenteral nutrition) (Fig. 1C) increased in all groups during the experiment although it remained lower in IR-JC rats. Consistent with their weight loss, plasma leptin and albumin levels were significantly lower in IR-JC and IR-JI groups than in the sham group (*P* < 0.05) (Fig. 1D,E),and tended to be lower for leptin in IR-JC PN group.

**Figure 2:**
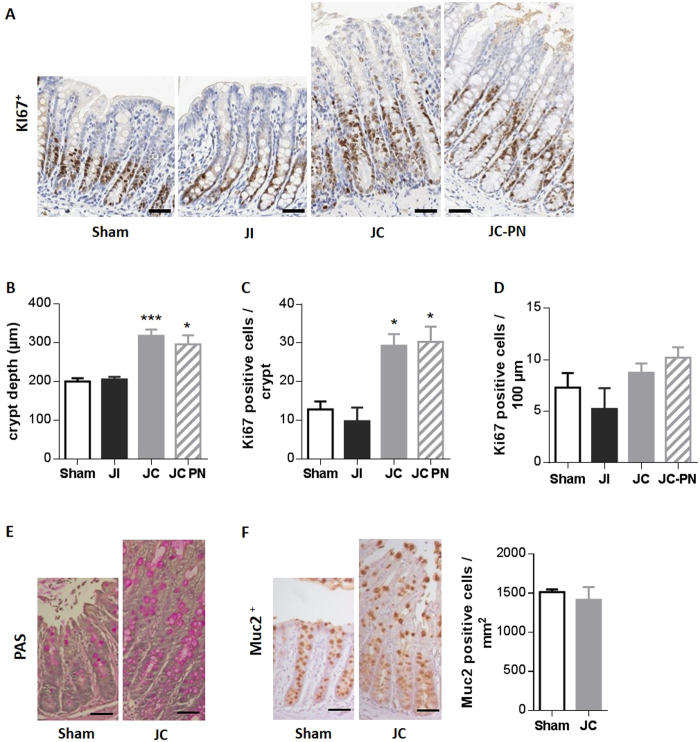
histological adaptation of the remaining colon mucosa after intestinal resection in rats. (**A**) Representative photomicrographs of colon mucosa of Sham, IR jejuno-ileal (JI), IR jejuno-colonic (JC) and IR jejuno-colonic with PN (JC-PN) rats immunostained with an anti-Ki67 antibody. (**B**) Measurement of colon mucosa crypt depth in μm (at least 5 crypts analyzed by rat). (**C**) Quantification of Ki67 positive cells per crypt (at least 5 crypts analyzed by rat), (**D**) density of Ki67 positive cells expressed as number of Ki67 positive cells per 100 μm of crypt. **(E,F**) Representative photomicrographs of colon mucosa of sham and IR-JC rats stained with Periodic Acid Schiff (PAS) (**E**) and Representative photomicrographs of colon mucosa of sham and IR-JC rats immunostained with anti-Muc2 antibody (**F**) with quantification of Muc2 positive cells per area in mm^2^. Data are represented as mean ± SEM of n = 6 for sham, n = 4 to 6 for IR jejuno-ileal, n = 10 for IR jejuno-colonic, n = 5 to 6 for for IR jejuno-colonic with PN. *P < 0.05, **P < 0.01, vs sham-operated rats based on non-parametric Kruskal-Wallis test followed by Dunn’s adjusted multiple comparisons. Scale bar: 50 μm.

Histological examination of the colon mucosa (Fig. 2A) showed that IR-JC rats had significantly deeper colonic crypts compared to sham rats (*P* < 0.001 IR JC vs sham and *P* < 0.05 IR JC-PN vs sham) (Fig. 2B), reflecting morphological adaptation. In addition they displayed higher number of Ki67-positive cells per crypt (Fig. 2A,C) (*P* < 0.05) than sham rats. On the contrary IR-JI rats were not significantly different from sham rats. The proliferative index was not significantly different between groups suggesting that the epithelial homeostasis was recovered at that post-operative time (Fig. 2D)

PAS mucin staining and Muc2 immunostaining of the residual colon mucosa of IR-JC rat, revealed an increase of mucus production compared to sham rat (Fig. 2E,F), as previously described for other preclinical models of large intestinal resection^26^. This greater number of goblet cells is probably a consequence of the increase of the crypt depth observed in IR-JC rats. The density of these cells was not modified in IR-JC compared to sham (Fig. 2F).

#### Higher plasma concentrations of gut hormones in rat models of SBS

**Figure 3:**
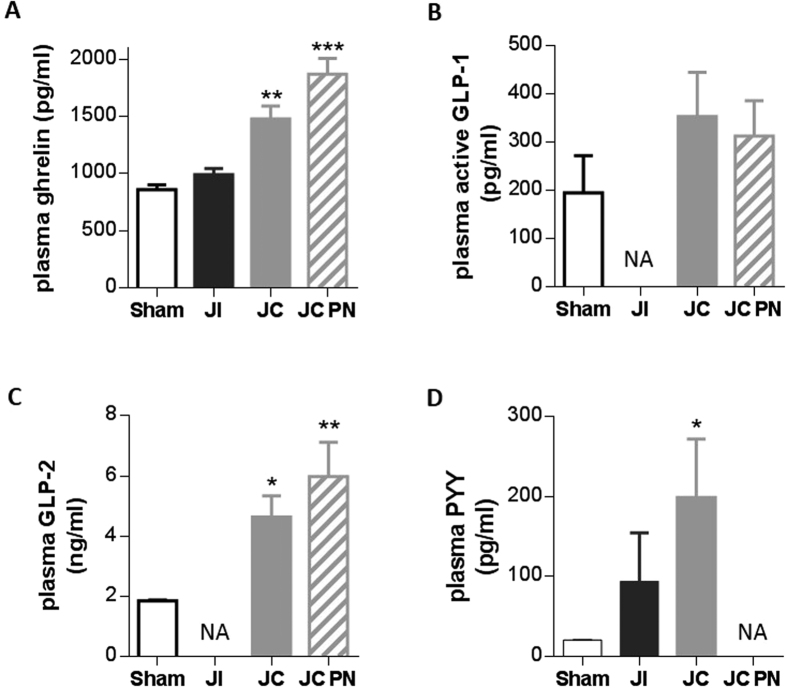
Increased levels of plasma gut hormones after intestinal resection in rats. Fasting plasma levels of ghrelin (**A**), GLP-1 (**B**) GLP-2 (**C**) and PYY (**D**) in IR jejuno-ileal (JI), IR jejuno-colonic (JC), IR jejuno-colonic with PN (JC-PN) and sham-operated (Sham) overnight fasted rats 7 days after surgery. Data are represented as mean ± SEM of n = 6 for sham, n = 4 or not available (NA) for IR-JI, n = 4 to 10 for IR JC, n = 4 to 6 or not available (NA) for IR-JC PN. *P < 0.05, **P < 0.01, vs sham-operated rats based on non-parametric Kruskal-Wallis test followed by Dunn’s adjusted multiple comparisons.

Fasting plasma ghrelin concentration was significantly higher in IR-JC rats with and without PN than in sham rats (*P* < 0.01) (Fig. 3A), whereas no such difference was observed in IR-JI rats. Fasting plasma GLP-1 and GLP-2 concentrations (Fig. 3B,C) were also higher in IR-JC rats with and without PN (*P* < 0.05 and < 0.01, respectively versus sham, for GLP-2). Fasting plasma PYY concentration, which was barely detectable in sham rats, was increased in IR-JI rats and was further enhanced in IR-JC rats (*P* < 0.05) (Fig. 3D). Unfortunately we were not able to determine plasma PYY in IR-JC PN rats and plasma GLP-1 and GLP-2 in IR-JI rats due to technical problems. However the higher plasma GLP-1, GLP-2 and PYY levels observed in overnight fasted rats were also found in non-fasted IR-JC rats (*P* < 0.01 or *P* < 0.05 vs sham) (Supplemental Figure S1).

#### No change in the densities of PYY and GLP-1 positive cells but higher levels of mRNA coding for proglucagon and PYY

**Figure 4:**
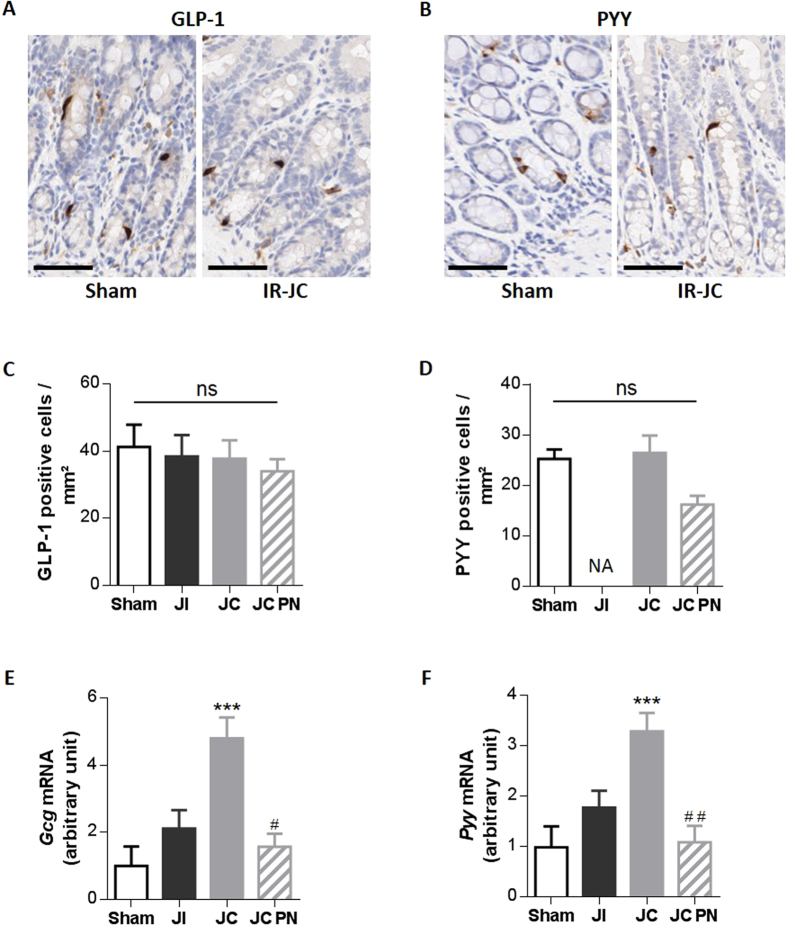
Unchanged number of PYY and GLP-1 positive cells but enhanced levels of *proglucagon* and *Pyy* mRNA after intestinal resection in rats. (**A,B**) Representative photomicrographs of GLP-1 (**A**) and PYY (**B**) immunostaining on colon mucosa sections from sham-operated and IR-JC rats (cytoplasm of GLP-1 or PYY cells are stained in brown), scale bar:50 μm. (**C,D**) Quantification of GLP-1 (**C**) and PYY (**D**) positive cells per area in mm^2^. Data are represented as mean ± SEM of n = 4 for sham, n = 4 or not available (NA) for IR jejuno-ileal (JI), n = 5 for IR jejuno-colonic (JC), n = 6 for for IR jejuno-colonic with PN (JC-PN). (**E–F**) Colonic mucosa mRNA levels of proglucagon (*Gcg*) (**E**) and *Pyy* (**F**) normalized to *L19*, 7 days after surgery. Data are represented as mean ± SEM of n = 6 for sham, n = 5 for IR-JI, n = 9 for IR-JC, n = 6 for IR-JC with PN. *P < 0.05, **P < 0.01, ***P < 0.001 vs sham-operated rats and ^#^P < 0.05 ^##^P < 0.01 vs IR-JC based on non-parametric Kruskal-Wallis test followed by Dunn’s adjusted multiple comparisons.

We investigated whether the elevation of these hormone levels was due to an increase in the number of enteroendocrine cells producing them and/or an increase in the hormone production by these cells. The density of GLP-1 positive cells was similar in the 3 groups of IR rats and in sham rats (Fig. 4A,C). The density of PYY-positive cells was unchanged in IR-JC rats without PN, and slightly lower in the colon mucosa of IR-JC rats with PN (Fig. 4B,D). Nevertheless, since the crypt depth is enhanced by 50% (Fig. 2A,B), this means that the total number of these cells will be increased similarly. The levels of mRNA coding for Gcg and PYY were 4 and 3-fold higher, respectively, in the colon mucosa of IR-JC rats than sham rats (*P* < 0.001) (Fig. 4E,F). IR-JC rats with PN had mRNA levels coding for Gcg and PYY similar to those observed in sham and IR-JI rats, but lower than those observed in IR-JC rats (*P* < 0.05).

#### Higher levels of mRNA coding for hypothalamic orexigenic neuropeptides (AgRP, NPY) and lower levels of mRNA coding for anorexigenic neuropeptides (POMC, CART) in rat models of SBS

**Figure 5:**
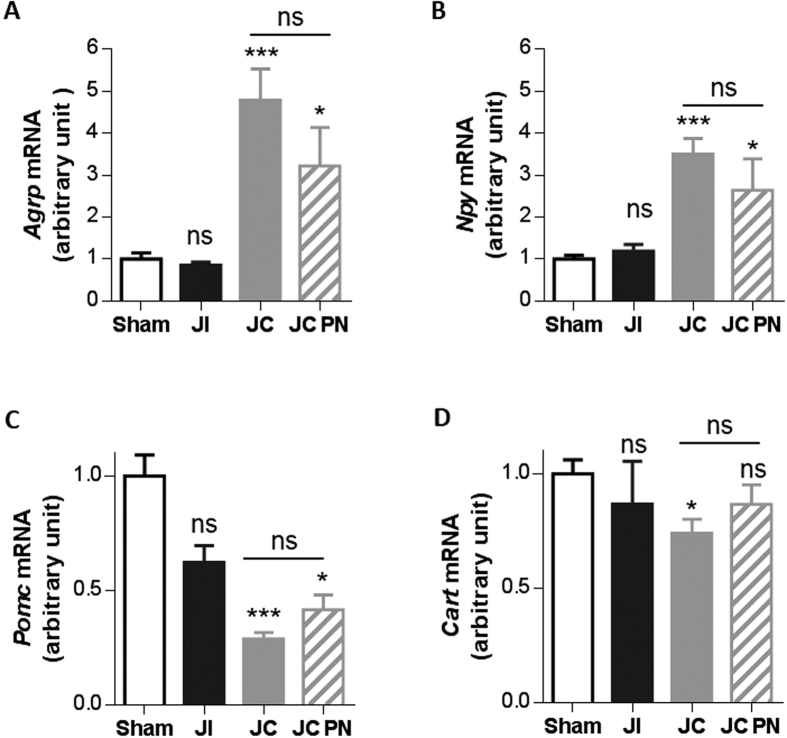
Expression of mRNA coding for hypothalamic orexigenic (AgRP, NPY) and anorexigenic (POMC, CART) neuropeptides after intestinal resection in rats. Expression of (**A**) *Agrp*, (**B**) *Npy*, (**C**) *Pomc* and (**D**) *Cart* mRNA normalized to *Hprt* mRNA in hypothalamus in IR jejuno-ileal (JI), IR jejuno-colonic (JC), IR jejuno-colonic with PN (JC-PN) and sham-operated (Sham) rats 7 days after surgery. Data are represented as mean ± SEM of n = 6 for sham, n = 5 for IR-JI, n = 10 for IR-JC, n = 6 for for IR-JC with PN.*P < 0.05, ***P < 0.001 vs sham-operated rats based on non-parametric Kruskal-Wallis test followed by Dunn’s adjusted multiple comparisons.

We investigated changes in the levels of hypothalamic neuropeptides after intestinal resection by analyzing mRNA levels for orexigenic (AgRP and NPY) and anorexigenic (CART and POMC) neuropeptides within the hypothalamus (Fig. 5).

IR-JC rats had significantly higher mRNA levels of AgRP and NPY than the sham rats (*P* < 0.001) (Fig. 5A,B). These levels were also significantly higher in IR-JC rats with PN than in sham rats (*P* < 0.05). Actually, mRNA levels of hypothalamic neuropeptides were not statistically different in IR-JC rats with PN or without PN.

Conversely, IR-JC rats (with or without PN) had significantly lower mRNA levels of POMC than the animals of the sham group (*P* < 0.001 and *P* < 0.05 respectively) (Fig. 5C,D). The decrease of mRNA levels coding CART was more subtle *(P* < 0.05). These results show that jejuno-colonic continuity induces substantial changes in the expression of hypothalamic neuropeptides controlling food intake and PN only slightly compensates this effect.

### Clinical study

#### Characteristics of SBS patients and control subjects

We studied SBS patients (medians for clinical characteristics are in Table 1) with jejuno-ileal (SBS-JI, *N* = 5) or jejuno-colonic (SBS-JC, *N* = 4) anastomosis. All SBS patients were considered to be in a steady state and had a remnant small bowel of less than 80 cm in length. All had normal albumin and prealbumin levels. Their concentration of plasma leptin was normal or low (Table 1). The average of total oral intake was 2271 (±288) kcal/day equivalent to 1.8 (±0.73) times their resting energy expenditure (REE). Six SBS patients were considered to have a high level of oral intake or hyperphagia^2^, i.e. with oral intake >1.5 time their REE (Table 1). Six of the SBS patients were dependent on PN (average of 1181 ± 787 kcal/perfusion). Patient’s gut hormone concentrations were compared with those of healthy subjects (C, *N = 5*) that were disease-free with normal eating behaviour.

#### Higher plasma concentrations of active ghrelin, GLP-1, GLP2 and PYY in SBS patients

**Figure 6:**
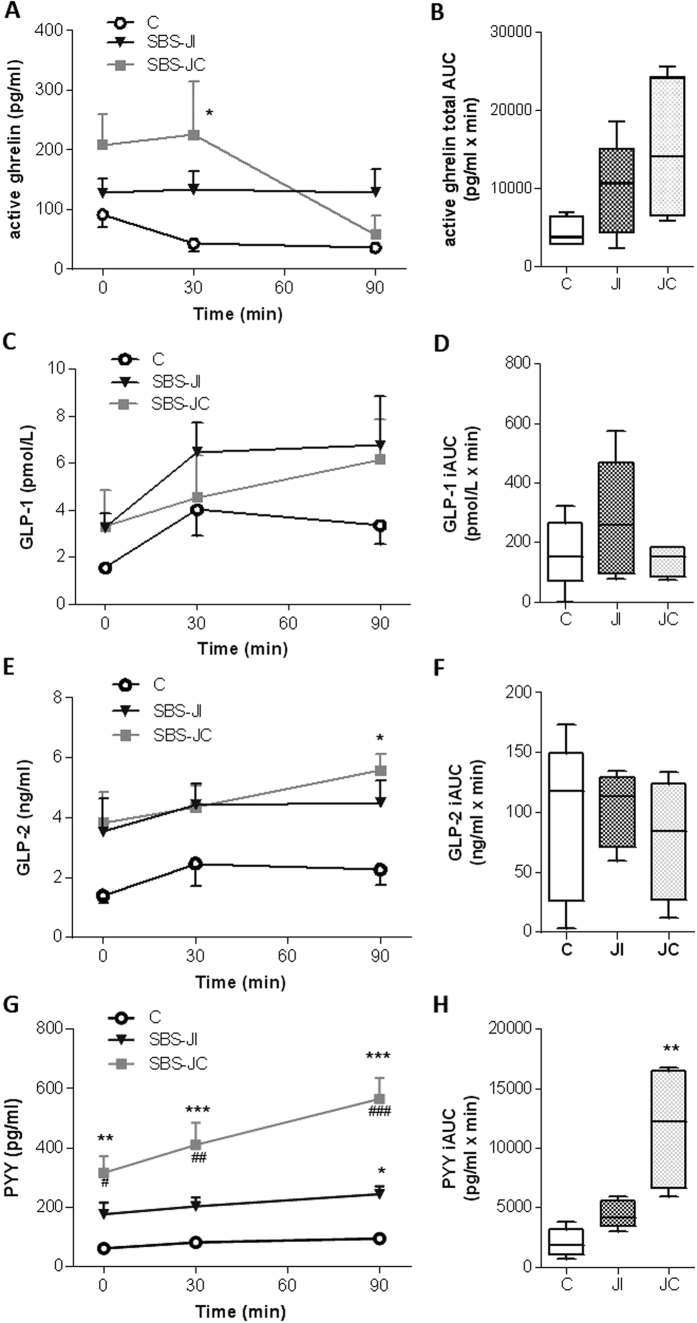
Increased fasting and post-prandial levels of plasma active Ghrelin, GLP-1, GLP2 and PYY in SBS patients. Plasma concentrations and secretory response to a calibrated meal (750 kcal) in SBS jejuno-ileal (JI) and SBS jejuno-colonic (JC) patients compared to healthy subjects (**C**) of: (**A,B**) active ghrelin (pg/mL) before (T0) and after (T30, T90) the meal (**A**) and ghrelin secretory response expressed as the total area under the curve (AUC) (**B**) GLP-1 (pg/mL) before (T0) and after (T30, T90) the meal (**C**) and GLP-1 secretory response expressed as the incremental area under the curve (iAUC) (**D**); (**E,F**) GLP-2 (pg/mL) before (T0) and after (T30, T90) the meal (**E**) and secretory response expressed as the iAUC (**F** PYY (pg/mL) before (T0) and after (T30, T90) the meal (**G**), and secretory response expressed as the iAUC (**H**). All values are expressed as the mean ± S.E.M of n = 5 for healthy subjects, n = 5 for jejuno-ileal SBS patients and n = 4 for jejuno-colonic SBS patients. *P < 0.05, **P < 0.01, ***P < 0.001 vs healthy subjects and ^#^P < 0.05, ^##^P < 0.01 and ^###^P < 0.001 vs jejuno-ileal SBS patients based for A, C, E, G, on Bonferroni’s multiple comparisons test and for B, D, F, H on non-parametric Kruskal-Wallis test followed by Dunn’s adjusted multiple comparisons.

As expected, active ghrelin levels decreased 30 minutes after eating the calibrated meal in control subjects, remaining low for at least 90 minutes (Supplemental Figure S2A). Fasting active ghrelin levels tended to be higher in SBS patients than in healthy controls (Supplemental Figure S2A),were significantly higher at 30 minutes in SBS patients (*P* < 0.05 vs controls) and decreased only at 90 minutes. The calculated total area under the curve (AUC), reflecting the absolute levels of ghrelin and the post-prandial response, was significantly greater (by a factor of two, *P* < 0.05 vs controls) in SBS patients (Supplemental Figure S2B). Distinguishing jejuno-colonic from jejuno-ileal SBS patients revealed that fasting active ghrelin elevation tended to be higher for jejuno-colonic SBS patients than for jejuno-ileal SBS patients and controls (Fig. 6A). Jejuno-colonic SBS patients exhibited a delayed meal-induced inhibition of plasma ghrelin concentration whereas no meal-induced inhibition of ghrelin concentration was observed in jejuno-ileal SBS patients (Fig. 6A).

Fasting plasma GLP-1 (3.286 ± 0.706 for SBS patients vs 1.553 ± 0.063 for controls, *P* < 0.05 in Mann-Whitney test) and GLP-2 (3.664 ± 0.716 for SBS patients vs 1.394 ± 0.252 for controls *P* < 0.05 in Mann-Whitney test) concentrations were both higher in SBS patients than in controls subjects (see also Supplemental Figure S2C,E). These increases were similar in jejuno-colonic and jejuno-ileal SBS patients (Fig. 6C,E). After the calibrated meal, the average plasma levels of these two hormones remained higher in SBS patients than in control subjects (Fig. 6C,E and supplemental Figure S2C,E), but the calculated incremental AUC (iAUC), reflecting the postprandial secretion response, did not differ significantly between patients and controls (Fig. 6D,F and Supplemental Figure S2D,F).

Fasting plasma PYY concentration was three times higher in SBS patients than in controls (*P* < 0.01 in Mann-Whitney tests) (Supplemental Figure S2G). This concentration increased significantly after a calibrated meal remaining higher in SBS patients than in controls. Accordingly, the calculated iAUC was significantly larger than that for controls (*P* < 0.01) (Supplemental Figure S2H). The difference in PYY levels (Fig. 6G) and PYY iAUC (*P* < 0.01) (Fig. 6H) with respect to controls was significantly greater in jejuno-colonic SBS patients than in those with jejuno-ileal anastomosis. Consistent with these observations, PYY levels were inversely correlated with the length of the remaining small bowel in SBS patients (Spearman coefficient: −0.76; *P* < 0.05). However, no correlations were found between circulating PYY, GLP-1 or ghrelin levels and daily oral intake or hyperphagic status in SBS patients.

## Discussion

This study is the first to demonstrate an increase of plasma ghrelin levels, major changes in the mRNA levels of hypothalamic orexigenic AgRP and NPY neuropeptides, and the elevation of PYY, together with GLP-2 and GLP-1, in rats with large intestinal resection including ileum, that are preclinical models of SBS. We also found that SBS patients had higher fasting and postprandial circulating ghrelin and PYY levels than controls, in particular patients with jejuno-colonic anastomosis, further emphazing the relevance of our preclinical model.

Since morphological and hormonal secretion adaptations have been reported to be maximal in different rat models of resection between day 4 and day 12 after intestinal resection^11^,^27^,^28^, the experimental period for the rat model was set up at 7 days. The colonic morphological adaptation observed in our IR-JC model are in agreement with previous reports for patient with jejuno-colonic anastomosis, i.e increase of crypt depth and of proliferating cell number with a controlled hyperplasia^29^ and increased GLP-2 secretions^3^.

Ghrelin is a well-known physiological hunger signal^30^,^31^ playing a major role in meal initiation^21^. Ghrelin-induced hunger involves the activation of central orexigenic pathways^32^ and stimulation of the expression of AgRP and NPY within the hypothalamus^24^,^33^,^34^. Higher levels of fasting ghrelin and of mRNA coding for orexigenic neuropeptides (AgRP and NPY) suggests a role for ghrelin in the activation of central orexigenic signals in IR jejuno-colonic rats. The lower levels of circulating leptin (an anorexigenic hormone) may also have contributed to the higher expression of the orexigenic neuropeptide^23^ in these IR rats. Of note, in a previous report^36^, and in our study, leptin concentrations in SBS patients was low but not different from controls. Furthermore, Molina *et al*. demonstrated that leptin concentrations did not correlate with hyperphagia in short bowel-patients^36^.

The central and peripheral variations of mRNA or peptide levels may also reflect the nutritional status of the rats. Ghrelin plays a role in long-term energy balance regulation, protecting against prolonged energy deficiency^32^. Indeed, prolonged starvation increases plasma ghrelin concentration in mice^37^ and may also influence NPY and AgRP mRNA levels^38^. We observed no hyperphagia in the IR rats during these 7 days of adaptation and all IR rats lost weight. Furthermore, the IR jejuno-colonic rats without PN, which are the leanest rats, had the highest levels of orexigenic signals suggesting that this may be an undernutrition response to promote food intake. IR jejuno-colonic rats with PN were heavier than jejuno-colonic rats without PN. Parenteral nutrition may ensure better hydration and improve energy balance by partially compensating for malabsorption, despite providing a moderate amount of calories. But, IR-JC rats with PN also exhibited significant increase in their orexigenic signals compared to IR jejuno-ileal rats even if these two groups had similar body weight. Overall, these observations suggest that the specific environment created by connecting the jejunum to the colon (including modifications of the lumen contents of nutrients, biliopancreatic secretions and microbiota metabolites) may trigger a specific activation of peripheral and central orexigenic cues beyond the known effect of undernutrition/starvation.

Evidence for the clinical relevance of these findings was provided by the study in human SBS patients. Indeed, these patients, who were not undernourished, had similar levels of leptin than healthy subjects and had high plasma concentrations of active ghrelin, with fasting and postprandial active ghrelin concentrations higher in jejuno-colonic SBS patients than in control individuals. In SBS patients, the postprandial inhibition of ghrelin levels was delayed, suggesting that this hunger signal persisted after the initiation of the meal in these patients. It remains unclear whether these persistently high ghrelin levels could induce hyperphagia in the long-term. Despite these differences, no relationships were found between ghrelin concentrations and the amount of ingested calories or the presence of hyperphagia, possibly because of the small number of SBS patients in this study. Further evaluation in a larger cohort of SBS patients would be required to unveil such relations.

The average concentrations of GLP-2, GLP-1 and PYY were systematically increased in IR rats, consistent with data reported for SBS patients (this study and^3^). Signals of hunger and satiety would be expected to operate in opposite ways. However, this does not appear to be the case here, given the concomitantly high concentrations of anorexigenic^14^ PYY and GLP-1 and orexigenic ghrelin found in IR rats and SBS patients. As suggested by others for GLP-1^39^,^40^, the high circulating concentrations of PYY, GLP-1 and GLP-2 may affect principally gastrointestinal tract functions rather than the central nervous system. In particular, these hormones decrease intestinal motility and transit time^18^, increasing the time for which nutrients are in contact with the remaining epithelium and thereby optimizing their distribution and absorption along the gastrointestinal tract. GLP-2 and PYY can also improve water and electrolyte absorption within the colon^41^,^42^. More specifically GLP-2 is also well known for its intestinotrophic effect^12^,^18^, and its increase could support, at least partially, the elevation of the colonic surface area we observed. Thus, this enhanced secretion of gut hormone reflects adaptive intestinal response, to improve overall digestion, absorption of water and nutrients and the epithelial surface available for this absorption.

The increase of fasting and postprandial secretion of PYY in SBS patients (this study and ref. 4) was larger in jejuno-colonic than in jejuno-ileal SBS patients and rats compared to control. No such differences were observed for GLP-1 and GLP-2 levels. Enteroendocrine cells are distributed from the duodenum to the colon but cells producing PYY are mostly restricted to the ileum and colon, and recently, it has been shown in rat that the highest concentrations of PYY along the gastrointestinal tract are found in the distal ileum and proximal and distal colon^43^. The higher PYY levels could result from an increase in the number of cells producing PYY due to morphological adaptation of the residual colon mucosa. The densities of PYY (and GLP-1) producing cells in colon were not affected in IR rats, suggesting that the homeostasis of epithelial cell differentiation was maintained in the colon of resected rats. But, the remaining colon mucosa in jejuno-colonic IR rats was hyperplasic with an enlarged number of proliferating cells and such changes were not observed in the colon mucosa when part of the ileum was preserved. A direct consequence of this colonic overgrowth would be a local increase in the number of enteroendocrine cells that in turn may contribute to the higher plasma concentrations of gut hormones. Alternatively, the colonic hormones-producing cells, and especially the PYY producing cells, may also have become highly responsive to the new environment created by the direct connection of the jejunum to the residual colon. The lumen of the residual colon typically contains large amounts of undigested nutrients^5^ and a dysbiotic microbiota^6^,^8^,^44^, and is thought to display changes in the fermentative activity generating short-chain fatty acids (SCFAs). SCFAs can modulate the function of enteroendocrine cells secreting GLP-1/GLP-2/PYY *in vitro*^45^,^46^ and possibly *in vivo*^46^,^47^. Bile acids could also participate to the increased colonic production of GLPs and PYY^48^,^49^, as well as to the increase number of proliferating cells^50^, observed in jejuno-colonic individuals. Whether the bile acids are present in different amount and composition in the colonic lumen content of the jejuno-ileal, jejuno-colonic and sham operated rat remain to be evaluated.

In conclusion, we demonstrate that SBS is associated with an increase in fasting plasma ghrelin concentration and an induction of orexigenic signals in the hypothalamus, in a rat model with jejuno-colonic anastomosis and that post-prandial ghrelin inhibition is impaired in SBS patients. The induction of peripheral gut hormones, such as PYY, is stronger in the absence of the ileum. Intestinal resection with jejuno-colonic anastomosis creates a specific environment promoting increase hunger signals and improving gastrointestinal functions associated with nutrient absorption. Further studies are required to determine whether these changes are correlated with the development of hyperphagia, a good prognosis for weaning off of PN.

## Materials and Methods

This study followed accepted ethical, scientific and medical standards and was conducted in accordance with recognized international standards, including the principles of the Declaration of Helsinki.

### Animal studies

All experimental procedures were performed in accordance with the European Community guidelines and were approved by and the Paris Nord local ethics committee and the Ministry of Education and Research (N°02285.01).

6-week-old male Wistar rats (n = 28) weighing 250–300 g (Elevage Janvier, Le Genest-St-Isle, France) were used. The animals were housed in cage with free access to standard rat chow diet (C1314 Altromin, Genestil, Royaucourt, France) and water, and maintained under standardized temperature (23 °C ± 1 °C) and 12 h light-dark cycles (lights on at 7:00 a.m.). Animals were allowed to acclimatize to their environment for 7 days before experimentation.

#### Surgical and experimental procedure

On the day of surgery, rats were randomly assigned to undergo resection of 70% of the small bowel with jejuno-ileal anastomosis (IR-JI), 80% of small bowel including the ileum, ileocecal valve and 20% of colon with jejuno-colonic anastomosis (for IR-JC), or a jejuno-jejunal transection with re-anastomosis (Sham) (Fig. 1A). Twelve hours before surgery, animals were fasted but allowed ad libitum access to water. They were anesthetized by intraperitoneal injection of pentobarbital sodium (Ceva, Libourne, France). Standard aseptic procedures were used throughout the surgery. Intestinal length was measured in a standardized method. The bowel was resected 15 cm distal to the ligament of Treitz to either 3 cm distal to the cecum for IR-JC rat groups or to 10 cm proximal to the cecum for IR JI rat groups and followed respectively by jejuno-colonic or jejuno-ileal anastomosis with 6–0 Prolene running sutures. Sham-operated rats underwent a jejuno-jejunal transection at 15 cm below the ligament of Treitz and re-anastomosed. Animals received subcutaneously Xylocaïne 1% (100 μl/100 g) (Astra, France) and Penicillin G (20,000 units/kg) (Panpharma, Luitre, France) to prevent postoperative pain and infections. Subcutaneous injection of 10 ml Bionolyte G5 (Sodium chloride [0.4%], Glucose [5.5%], Potassium Chloride [0.2%]) (Baxter, Maurepas, France) was given to prevent postoperative dehydration. Liquid diet (Nutrison, Nutricia, France) was available *ad libitum* 48 h after surgery. The IR jejuno-colonic rats were under oral nutrition without or with PN through a catheter implanted in the jugular vein. PN diet, prepared aseptically, was perfused 48 h after surgery via a syringe pump (Precidor-Infors AG Basel) at 1.5 ml/h during 8 h daily, providing 12 kcal/day. Free access to food and PN administration were restored 48 hours after surgery and total calorie intake was calculated daily.

Previous studies have reported maximal intestinal morphological changes between day 4 and 12 after the surgery^11^,^27^.Thus, we set 7 days as the experimental period for this study. Body weight was monitored daily and food intake was determined from day 2 to 7 post-surgery. At day 7, overnight fasted rats were anesthetized; blood was collected in chilled tubes containing heparin or heparin and dipeptidyl peptidase IV (DPP-IV) inhibitor (Roche Applied Science). Plasma was aliquoted and stored in −80 °C until analyses. Then the rats were sacrificed, hypothalamus and intestinal mucosa scrappings were collected and rapidly frozen until extraction of total ARN for RTq-PCR analyses. Other intestinal segments were formalin-fixed overnight and embedded in paraffin for classical histology and immunohistochemistry studies (*see* Supplementary Material).

#### Quantification of gene expression in colon mucosa and hypothalamus

Total RNA was extracted from unfrozen colon mucosa scrappings and from hypothalamus tissue with Trizol reagent (Invitrogen, Saint Aubin, France). After reverse transcription with 8 μg total RNA, RTq-PCR real-time qPCR was performed in duplicate for each cDNA, using specific primers and the ABI PRISM 7000 sequence detection system and TaqMan universal PCR technology as described in details in Supplementary Material and compared to mean target gene expression of sham operated rats as calibrator samples. Fold induction was calculated using the comparative 2^−Δct^ Method.

### Clinical study

#### Human subjects

Nine adult patients with SBS were recruited from our tertiary care center for intestinal failure (Beaujon Hospital). They gave informed consent for participation to the study. The protocol was approved (No. 15–046) by the Institutional Review Board-IRB-00006477- of HUPNVS, Paris 7 University, APHP.

The patients were in a steady state, with their last surgery realized more than 6 months before the blood test. They all were on a non-restricted oral diet and 6 of them had PN dependence. Criteria for inclusion were extensive small bowel resection, with a remnant small bowel length <150 cm, with some colon in continuity (jeuno-colonic anastomosis) or with ileum in continuity (jeuno-ileal anastomosis). Criteria for exclusion were upper gastrointestinal tract surgery, small bowel lesions in the remnant gut, organ failure other than digestive, evolutive neoplasia, intestinal fistulae and treatment by growth hormone, GLP-2, steroids or immunosuppressive therapy in the 4 months preceding the study. The patients had no signs of sepsis and no antibiotherapy.

Five adult healthy subjects with normal BMI (between 18.5–24.9 kg/m^2^) who gave their informed consent for the study were also recruited (ANSM authorization B131095–81). Procedures followed were in accordance with the ethical standards of the Helsinki Declaration of 1975, as revised in 1983.

#### Study design

The blood samples were collected in SBS and control subjects during their routine monitoring after an overnight fast (SBS on PN receiving saline solution to avoid dehydration), before (T0), 30 and 90 min after consumption of a standardized breakfast of 725 kcal (100 g of bread, 20 g of butter, 20 g of jam, 30 g of French emmental, 20 g of powder skimmed milk, 10 g of sugar, 250 ml of tea or coffee and 500 ml of Vichy St-Yorre water). Blood samples were drawn in EDTA tube Vacutainer (Becton Dickinson, France) containing DPP-IV inhibitor (DPP-IV-010, Millipore) and serine protease inhibitor, 4-(Aminoethyl)-benzenesulfolnyl fluoride hydrochloride or Pefabloc (Roche Applied Science). Samples were centrifuged between 863 to 2000 g for 10 min. Aliquoted plasma was stored in −80 °C until analyses.

### Plasma hormone analyses

Human and rat plasma concentrations of PYY, GLP-1, GLP-2 and ghrelin were quantified as described in details (Supplementary Material). The hormone assessment results were expressed as absolute value and incremental area under the curve (iAUC) calculated by subtracting baseline levels from all subsequent readings or total area under the curve (tAUC) for ghrelin.

### Statistical analyses

All values are expressed as the mean ± S.E.M. Non-parametric tests were used: Mann-Whitney test to compare two groups and Kruskal-Wallis test followed by Dunn’s adjusted multiple comparisons to compare more than two groups were performed using GraphPad Prism version 6.0 for Windows (GraphPad Software, San Diego, CA, USA). A value of *P* < 0.05 was considered statistically significant.

## Additional Information

**How to cite this article**: Gillard, L. *et al*. Enhanced Ghrelin Levels and Hypothalamic Orexigenic AgRP and NPY Neuropeptide Expression in Models of Jejuno-Colonic Short Bowel Syndrome. *Sci. Rep.* **6**, 28345; doi: 10.1038/srep28345 (2016).

## Supplementary Material

Supplementary Information

## Figures and Tables

**Table 1:** Clinical and nutritional characteristics of SBS patients.

SBS patients (sex)	Age	BMI	Remnant jejunum/ ileum length (cm)	Remnant colon (%)	Delay since continuity restablishment (months)	Total oral intake (kcal/day)	PN/Week (n)	PN support (kcal/perfusion)	REE (kcal/day)	Oral intake /REE	Leptin (ng/ml)	Prealbumin (g/l)
*P1 (M)*	50	22	10/40 (SBS-JI)	100	12	3000	2	1612	1442	2,1	2,2	0,305
*P2 (F)*	59	16	20/0 (SBS-JC)	75	132	2200	4	2450	1070	2,1	2,2	0,194
*P3 (F)*	52	20	20/50 (SBS-JI)	100	24	2200	0	0	1188	1,9	1,99	0,163
*P4 (F)*	49	19	12/10 (SBS-JI)	100	96	3600	3	1498	1192	3,0	3,09	0,221
*P5 (F)*	39	23	70/5 (SBS-JI)	100	60	1500	0	0	1419	1,1	14,5	0,187
*P6 (F)*	32	19	0/0 (SBS-JC)	50	12	840	7	1910	1348	0,6	2,71	0,313
*P7 (M)*	73	20	10/40 (SBS-JI)	100	96	3000	0	0	1214	2,5	1,99	0,263
*P8 (M)*	57	19	15/0 (SBS-JC)	50	120	1600	5	1662	1195	1,3	4,56	0,289
*P9 (F)*	63	25	80/0 (SBS-JC)	50	12	2500	2	1498	1307	1,9	43,18	0,288
***Median*** [25–75]	**52** [49–59]	**20** [19–22]	**15** [10–45]/**5** [0–40]	**100** [50–100]	**60** [12–96]	**2200** [1600–3000]	**2** [0–4]	**1498** [0–1662]	**1214** [1192–1348]	**1,9** [1,1]	**2,71** [2,2–4.56]	**0,276** [0,194–0,289]

BMI: body mass index (Kg/m2). SBS-JI = SBS patients with jejuno-ileal anastomosis, SBS-JC = SBS patients with jejuno-colonic anastomosis. Remnant jejunum and ileum length and colon in continuity were expressed in cm and in % of common colon length, respectively. Delay was in months from the last surgery establishing an anastomosis between the jejunum and the remaining colon. Total oral was expressed in Kcal/day. Parenteral nutrition (PN) support was expressed in number (n) of perfusion per week (PN/week) and kcal/perfusion. Resting energy expenditure (REE) was expressed in kcal/day. Hyperphagia is defined as oral intake/REE > 1.5. Plasma levels of leptin are in ng/mL and prealbumin are in g/L. Values are median and 25th and 75th percentile. Etiology of SBS were arterial mesenteric infraction (n = 5), volvulus (n = 1), intestinal occlusion (n = 1) and surgical complications (n = 3) with no residual lesions of the remnant intestine.
