# Supplementary material for: Enhanced Ghrelin Levels and Hypothalamic Orexigenic AgRP and NPY Neuropeptide Expression in Models of Jejuno-Colonic Short Bowel Syndrome
Source: Sci Rep. 2016 Jun 21;6:28345. doi: 10.1038/srep28345 (PMC4914859; doi:10.1038/srep28345)

SUPPLEMENTARY INFORMATION

Enhanced Ghrelin Levels and Hypothalamic Orexigenic AgRP and NPY Neuropeptides Expression in Models of Jejuno-Colonic Short Bowel Syndrome

Laura GILLARD[1], Lore BILLIAUWS[1,2], Bogdan STAN-IUGA[1], Lara RIBEIRO-PARENTI[1,3], Anne-Charlotte JARRY[1], Jean-Baptiste CAVIN[1], Françoise CLUZEAUD[1], Camille MAYEUR[4], Muriel THOMAS[4] , Jean-Noël FREUND[5], Jean-Marc LACORTE[6,7,8], Maude LE GALL[1], André BADO[1], Francisca JOLY[1,2, $,*], Johanne LE BEYEC[1,7,8, $,*]

SUPPLEMENTARY MATERIALS AND METHODS

Histology and immunohistochemistry studies. Colon segments were fixed overnight in 10% neutral buffered formalin, paraffin-embedded, and sectioned at 5 µm. The sections were stained with hematoxylin phloxine saffron (HPS) or immunostained with rat monoclonal anti-Ki67 antibody (M724801 clone MIB-5, RUO, DAKO, Les Ulis, France), diluted 1/25; anti-Muc2 (Santa Cruz Biotechnology Inc), diluted 1/1000; anti- GLP-1 antibody (ab26278, Abcam®), diluted 1/3000; or anti-PYY antibody (ab131246, Abcam®), diluted 1/200. Immunohistochemistry was carried out using an automated immunohistochemical stainer (Bond-Max autostainer, Leica, Wetzlar, Germany). Each slide was scanned with Aperio ScanScope CS System (Leica Microsystemes SAS, Nanterre France). The images were analyzed with TRIBVN CaloPix software (TRIBVN, Chatillon France).

RNA extraction and qPCR analyses. Total RNA was extracted from colon mucosa scrapping and from hypothalamus tissue with Trizol reagent (Invitrogen, Saint Aubin, France). After The RNA quality was determined by Agilent 2100 Bioanalyser (Agilent Technologies, Santa Clara, CA, USA). Reverse transcription was performed with 8µg total RNA using a high-capacity cDNA reverse transcription kit (Applied Biosystems). Real-time qPCR was performed in duplicate for each cDNA, using specific primers and the ABI PRISM 7000 sequence detection system and TaqMan universal PCR technology. Primers were designed by Applied Biosystems for *Gcg* (Rn00562293_m1) and for *Pyy* (Rn01460420_g1). The Light Cycler detection system (Roche Diagnostics) was used to quantify hypothalamic cDNA for *Agrp* (primers forward:

cagagttctcaggtctaagtc and reverse: ttgaagaagcggcagtagcac), *Npy* (primers forward: ccgctctgcgacactacatand reverse: tgtctcagggctggatctct), *Pomc* (primers forward: aggacctcaccacggaaag and reverse: ccgagaggtcgagtctgc) and *Cart* (primers forward: tacggccaagtccccatgtgand reverse: ggggaacgcaaactttattgttg). Results were normalized to ribosomal RNA *L19* levels (primers forward: tgccggaagaacaccttg, and reverse: gcaggatcctcatccttcg) for colon mucosa and to *Hprt* levels (primers forward: gaccggttctgtcatgtcg, and reverse: acctggttcatcactaatcac) for hypothalamus Fold induction was calculated using the comparative $2^{-\Delta ct}$ Method.

Energy intake of SBS patients. Ingesta were evaluated by a trained dietician. Oral intake was unrestricted for 3 days during the week preceding samples collection and was estimated by dietary questionnaire as the mean of the oral intake during these three days. The daily intake of total calories, were calculated with bilnut® software Hyperphagia was defined as oral intake 1.5 times > to resting energy expenditure (REE), according to Harris-Benedict equations[5]. Parenteral nutrition was defined by the number of parenteral support infusions (0-7) per week and by the total energy per infusion (Kcal/infusion)

Plasma hormone analyses. Human plasma concentrations of PYY and active ghrelin were quantified on a Luminex Magpix 200® analyser using a Millipore/miliplex human gut hormone panel (HGT-68K-03, Merck Millipore, Saint-Quentin en Yvelines, France) with reported intra- and inter-assay coefficients of variation < to 11% and 19% respectively. Human GLP-1 was measured by ELISA (EDI™ Total GLP-1 ELISA kit, KT 876, Epitope diagnostics, France) with reported intra- and inter-assay coefficients of variation < to 4.7% and < to 9.5% respectively. Human and rat GLP-2 levels were determined by ELISA (EZGLP2-37K, Merck Millipore, Saint-Quentin en Yvelines, France) with reported intra- and inter-assay coefficients of variation < to 9.1% and < to 11.5% respectively. Rat GLP-1, PYY and leptin were quantified on a Luminex Magpix 200® analyser using a Millipore/miliplex rat gut hormone panel (# RMHMAG-84K, Merck Millipore, Saint-Quentin en Yvelines, France) with reported intra- and inter-assay coefficients of

variation of 3% and < 11% respectively. Total ghrelin of rat was measured by RIA (MI-GHRT-89HK Merck Millipore, Saint-Quentin en Yvelines, France) with reported intra- and inter-assay coefficients of variation < to 10% and < to 16.7% respectively. The results of hormone measurements were expressed as absolute value and total or incremental area under the curve (AUC), calculated by subtracting baseline levels from all subsequent readings.

SUPPLEMENTAL FIGURE LEGENDS

Supplemental figure S1:

Increased GLP-1 and GLP-2 Plasma levels in fed Sham and IR-JC rat models: Fold increase of plasma levels of GLP-1 (**A**), GLP-2 (**B**) and PYY (**C**) in non-fasted IR jejuno-colonic (JC) compared to sham-operated (Sham) rats with free access to food 7 days after surgery. Data are represented as mean ± SEM of n=6 for sham, n=10 for IR jejuno-colonic. *P<0.05, **P<0.01, vs sham-operated rats based on Mann-Whitney test.

Supplemental figure S2:

Increased fasted and post-prandial levels of plasma active Ghrelin, GLP-1, GLP2 and PYY in SBS patients : Plasma concentrations and secretory response to a calibrated meal (750 Kcal) in all SBS (purple) patients compared to healthy subjects (C) (black) of : (**A-B**) active ghrelin (pg/ml) before (T0) and after (T30, T90) the meal (**A**), and ghrelin secretory response expressed as the total area under the curve (AUC) (**B**) ; (**C-D**) GLP-1 (pmol/L) before (T0) and after (T30, T90) the meal (**C**) and GLP-1 secretory response expressed as the incremental area under the curve (iAUC) (**D**) ; (**E-F**) GLP-2 (pmol/L) concentration before (T0) and after (T30, T90) the meal (**E**) and secretory response expressed as the iAUC (**F**) ; (**G-H**) PYY (pg/ml) concentration before (T0) and after (T30, T90) the meal (**G**) and secretory response expressed as iAUC (**H**). All values are expressed as the mean ± SEM of n=5 for healthy subjects and n= 9 for SBS. *P<0.05, **P<0.01, ***P<0.001 based for A, C, E, G, on Bonferroni's multiple comparisons test and for B, D, F, H on non-parametric Kruskal-Wallis test followed by Dunn’s adjusted multiple comparisons.

SUPPLEMENTAL FIGURE S1

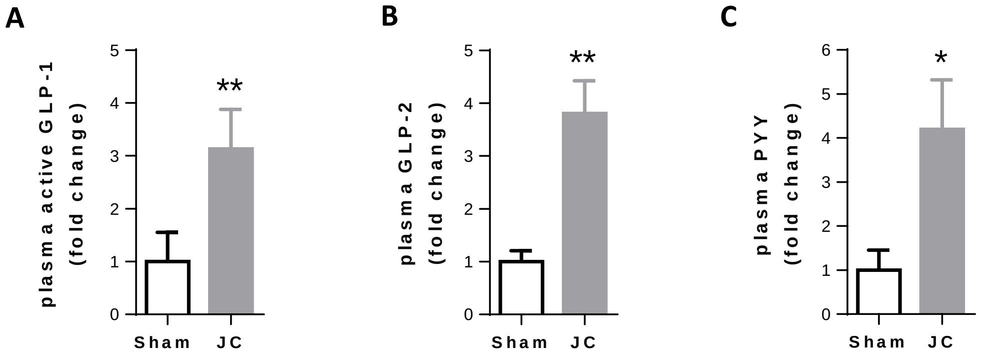

SUPPLEMENTAL FIGURE S2

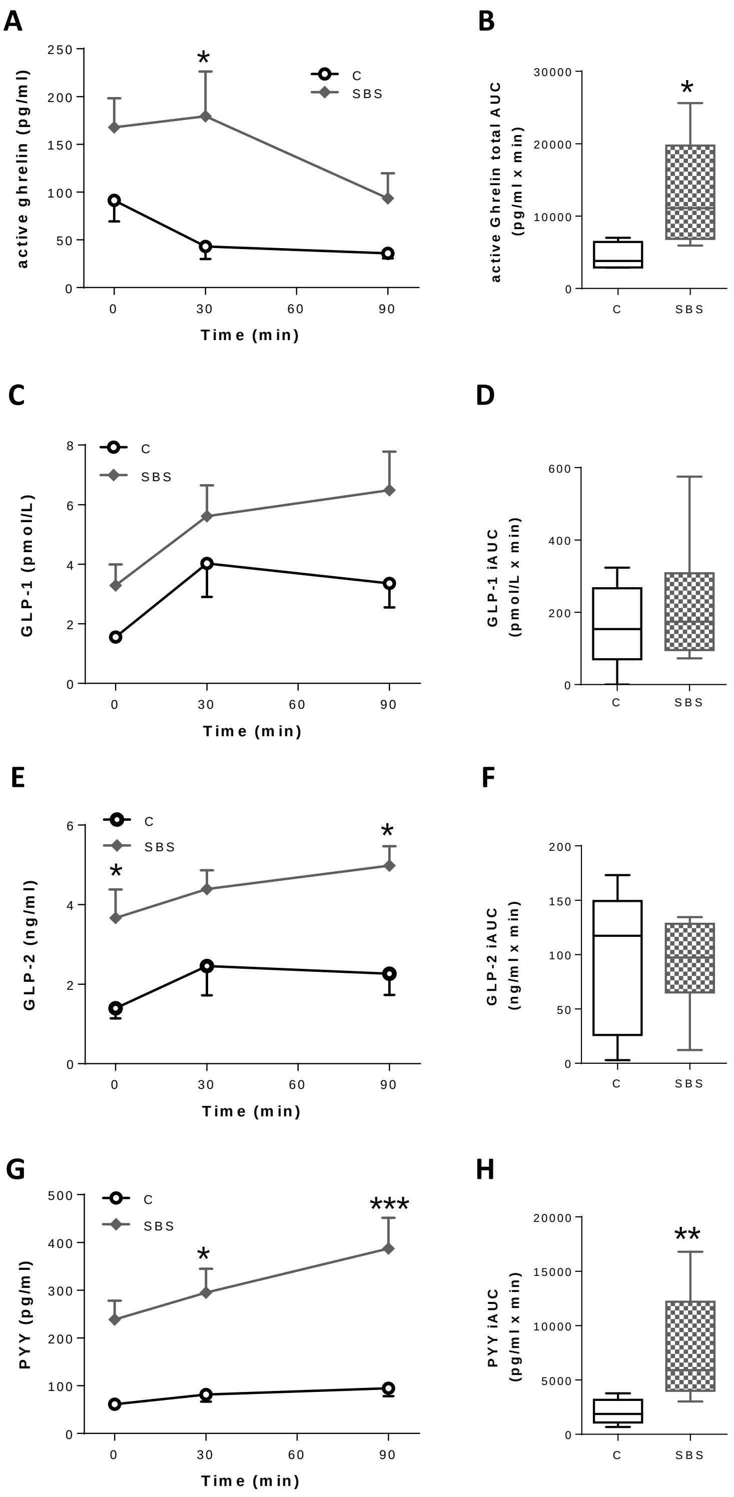

Supplement: Supplementary Information [file srep28345-s1.pdf]
